# Bibliometric analysis of the inflammation in diabetic cardiomyopathy

**DOI:** 10.3389/fcvm.2022.1006213

**Published:** 2022-12-13

**Authors:** Ning Zhu, Bingwu Huang, Liuyan Zhu

**Affiliations:** ^1^Department of Cardiology, The Third Affiliated Hospital of Shanghai University, Wenzhou People’s Hospital, The Wenzhou Third Clinical Institute Affiliated to Wenzhou Medical University, Wenzhou, China; ^2^Department of Anesthesiology and Perioperative Medicine, The Second Affiliated Hospital and Yuying Children’s Hospital of Wenzhou Medical University, Wenzhou, China; ^3^Department of General Practice, The Third Affiliated Hospital of Shanghai University, Wenzhou People’s Hospital, The Wenzhou Third Clinical Institute Affiliated to Wenzhou Medical University, Wenzhou, China

**Keywords:** inflammation, diabetic cardiomyopathy, bibliometric analysis, VOSviewer, Web of Science

## Abstract

**Background:**

Maladaptive inflammation is implicated in the development of diabetic cardiomyopathy (DCM). This study aimed to visually analyze the global scientific output over the past two decades regarding research on inflammation associated with DCM.

**Methods:**

All relevant articles and reviews were retrieved in the Web of Science (WOS) Core Collection (limited to SCIE) using “inflammation” and “diabetic cardiomyopathy” as search terms. Articles and reviews published from 1 January 2001 to 28 February 2021 were collected. Visualization analysis and statistical analysis were conducted by Microsoft 365 Excel and VOSviewer 1.6.18.

**Results:**

A total of 578 documents were finally selected for further analysis. The publications regarding inflammation and DCM increased gradually over approximately 20 years. The most prolific country was China, with 296 documents and the most citations (9,366). The most influential author groups were Lu Cai and Yihui Tan who were from the United States. The bibliometric analysis of co-occurrence keywords showed that inflammation in DCM is composed of numerous molecules (NF-κB, NLRP3 inflammasome, Nrf-2, TNF-α, protein kinase C, PPARα, TLR4, p38 mitogen-activated protein kinase, TGF-β, Sirt1, and AKT), a variety of cardiac cell types (stem cell, fibroblast, and cardiomyocyte), physiological processes (apoptosis, oxidative stress, autophagy, endoplasmic reticulum stress, hypertrophy, mitochondrion dysfunction, and proliferation), and drugs (sulforaphane, metformin, empagliflozin, and rosuvastatin).

**Conclusion:**

Our bibliometric analysis presents the characteristics and trends of inflammation in DCM and shows that research on inflammation in DCM will continue to be a hotspot.

## Introduction

Diabetic cardiomyopathy (DCM) is a disease that is characterized by structure abnormality and cardiac dysfunctions but in the absence of coronary artery disease, valvular diseases, and other conventional cardiovascular risk factors (1). DCM is now rapidly increasing with the increased prevalence of diabetes. The development and progression of DCM are associated with multiple factors, including impaired cardiac insulin metabolic signaling, extracellular matrix stiffness, endoplasmic reticulum stress, mitochondrial dysfunction, oxidative stress, and microvascular dysfunction (2). Furthermore, it has been demonstrated that hyperglycemia-induced cumulative inflammation is characteristic of the initial stage of DCM (1). Emerging evidence suggests that targeting inflammation is promising for ameliorating DCM, although little has been known regarding the underlying mechanisms (3).

Bibliometrics, a widely accepted research method, can build up the knowledge structure and track developmental trends by statistically and visually analyzing a specific research field over a certain time frame (4, 5). Bibliometrics analysis compares contributions among key information on countries, institutions, journals, authors, and their literature references from the Web of Science (WOS) Core Collection database (6). Several bibliometric software, such as VOSviewer, CiteSpace, and BibExcel, have been developed to conduct bibliometrics analysis frequently (5, 7, 8). Bibliometrics has been widely used in many research areas such as infectious diseases, energy utilization, environmental science and pollution, and health service (9–12). In addition, bibliometrics has been increasingly prevalent in applications for governing and improving clinical and research guidelines and policy making, as well as research hotspots and trends (13–16).

In the past two decades, a large number of studies focusing on the role of inflammation in DCM have been published. However, from the perspective of bibliometrics, few papers provided an overview of inflammation related to DCM. Hence, this study applied bibliometric analysis to comprehensively investigate the research trends concerning inflammation related to DCM from the aspects of key journals and authors, important cited references, number of publications per year, and most productive countries/regions. This study aimed to illustrate the research landscape and trends for clinical researchers and practitioners.

## Materials and methods

### Data sources and search strategy

All data were retrieved from the Web of Science (WOS) Core Collection, including the Science Citation Index Expanded with a publication timespan (1 January 2001 to 28 February 2021). These data were composed of full records and cited references. The search terms were “inflammation” and “diabetic cardiomyopathy.” The document types were restricted to articles or reviews. We reviewed the abstracts or the full text of the controversial publications. Publications that were unrelated to the search topic were excluded.

### Bibliometric analysis and visualization

We used Microsoft 365 Excel to assess and visualize the number of publications among different countries. VOSviewer 1.6.18 was used to analyze and illustrate the top journals, authors, their institutions, publication quantity, and keywords co-occurrence, as well as cited references. The parameters of the VOSviewer were set as follows: Linlog/modularity and full counting. VOSviewer is a software tool for constructing and viewing bibliometric maps, including co-occurrence, co-authorship, keyword co-occurrence, citation, bibliographic coupling, and co-citation. The “citation report” function from WOS was also applied to determine citation rates and the h-index of authors.

## Results

### General statistics

**FIGURE 1:**
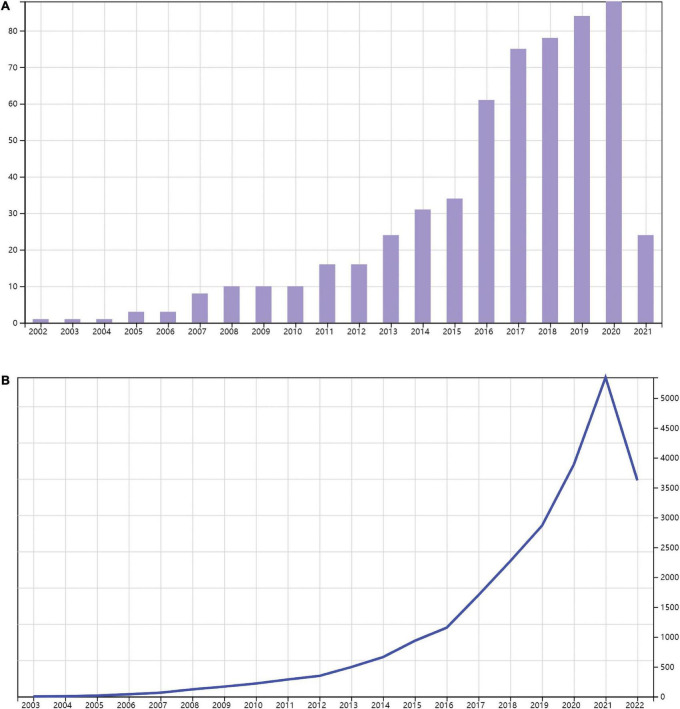
Trends in publications of inflammation related to diabetic cardiomyopathy (DCM) research. Timeline of publications **(A)** and citations **(B)** on inflammation related to DCM.

A total of 682 publications meet the criterion. The articles that were not related to inflammation or DCM (*n* = 104) were excluded. Finally, 578 papers satisfying these search criteria were extracted for further analysis. As is shown in Figure 1, 457 articles and 121 reviews on inflammation related to DCM in the WOS Core Collection from 1 January 2001 to 28 February 2021 were identified. The majority of these articles were written in English (574/99.3%), with the rest being in Japanese, Polish, Russian, and Spanish. The result showed that the publications of inflammation in DCM were of sustained growth every year over the studied period. Accordingly, the total number of citations rapidly increased in line with the number of publications. The publication number and citation numbers were the highest in 2020. These data suggested the growing interest in the research of inflammation in DCM in the past two decades.

### Countries/regions

**FIGURE 2:**
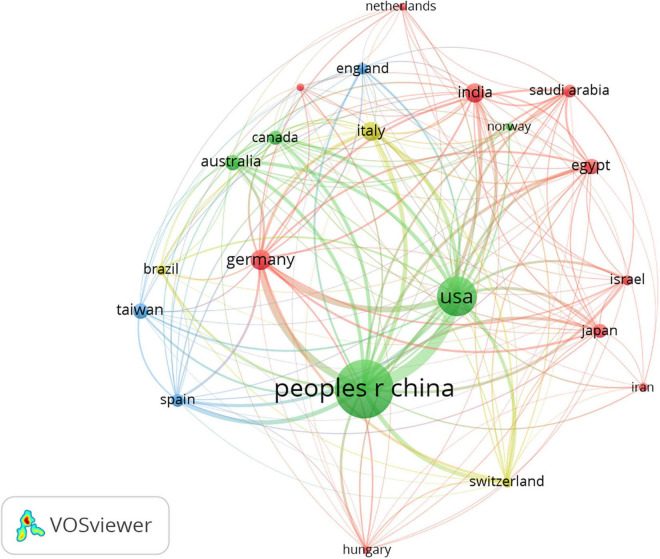
Network map of countries/regions in inflammation related to diabetic cardiomyopathy (DCM) research. The analysis method was Linlog/modularity in VOSviewer, the weight was an occurrence, and scores were the average published year.

A total of 55 countries/regions contributed to the research of inflammation in DCM, and 21 satisfied the criterion (minimum number of documents of a keyword: 5). Figure 2 shows the global distribution of all the analyzed articles, and Table 1 shows the 10 most productive countries/regions. China published the highest number of papers (*n* = 296), followed by the United States (*n* = 135), Germany (*n* = 36), Italy (*n* = 35), and India (*n* = 33). China had the highest number of citations (*n* = 9,366) followed by the United States (*n* = 8,835), Germany (*n* = 3,027), Italy (*n* = 1,548), and India (*n* = 1,309). China had the closest cooperation with the United States.

**TABLE 1:** Top 10 the most productive countries/regions on inflammation in diabetic cardiomyopathy (DCM).

Rank	Countries/regions	Documents	Citations
1	China	296	9,366
2	United States	135	8,835
3	Germany	36	3,027
4	Italy	35	1,548
5	India	33	1,755
6	Australia	23	941
7	Egypt	21	583
8	Taiwan	21	590
9	Japan	19	968
10	Canada	18	1,081

### Journals

According to the statistical analysis, the top 10 published journals are shown in Table 2. Impact factor (IF) and Journal Citation Reports partition were identified. The most published and popular journals were Biomedicine & Pharmacotherapy (*n* = 16, IF 2021 = 7.419, Q1) and Diabetes (*n* = 16, IF 2021 = 9.337, Q1). Four journals were from the United States, two journals were issued by the United Kingdom, two journals were issued by Switzerland, and others were issued by Egypt, Germany, and France. The 2021 impact factor of these journals ranged from 3.752 to 10.406. Diabetologia had the highest IF, while PLoS One had the lowest IF. The JCR partition analysis showed that six journals were Q1 and the other four were Q2.

**TABLE 2:** Top 10 journals with the largest number of publications on inflammation in DCM.

Rank	Journals	Documents	2019 impact factor	2019 JCR partition
1	Biomedicine & Pharmacotherapy	16	7.419	Q1
2	Diabetes	16	9.337	Q1
3	Oxidative Medicine and Cellular Longevity	15	7.310	Q2
4	Cardiovascular Diabetology	14	7.332	Q2
5	PLoS One	13	3.752	Q2
6	International Journal of Molecular Sciences	12	6.208	Q1
7	Journal of Cellular and Molecular Medicine	12	5.295	Q2
8	American Journal of Physiology-Heart and Circulatory Physiology	11	5.125	Q1
9	Diabetologia	10	10.406	Q1
10	Frontiers in Physiology	10	4.755	Q1

### Organizations

**FIGURE 3:**
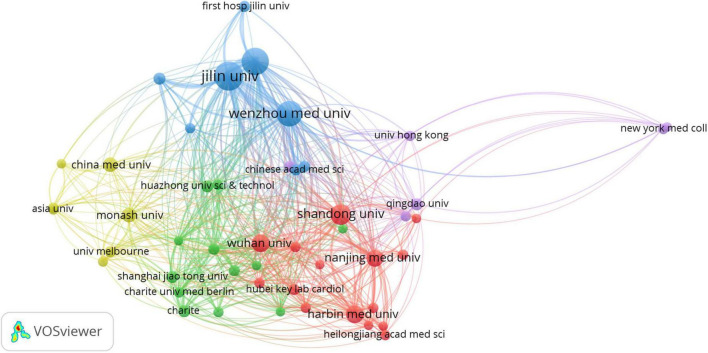
Network map of co-author analysis of organizations. The method was Linlog/modularity, weight was citations, and scores were the average published year. The analysis method was Linlog/modularity in VOSviewer, the weight was an occurrence, and scores were the average published year.

According to VOSviewer analysis, 578 documents were published by 766 organizations, and 48 satisfied the criterion (minimum number of documents of a keyword: 5). After excluding disjointed organizations, the remaining 48 organizations were constructed for the visualization map. The network map of co-author analysis of organizations is constructed in Figure 3 and the top 10 most productive organizations are listed in Table 3, including eight Chinese organizations and two United States organizations. Jilin University (*n* = 44) and Wenzhou Medical University (*n* = 43) had larger-sized bubbles representing higher numbers of papers. The number of citations from Jilin University (*n* = 2,019) was also more than that from Wenzhou Medical University (*n* = 1,966). Jilin University had the closest cooperation with the University of Louisville.

**TABLE 3:** Top 10 the most productive organizations on inflammation in diabetic cardiomyopathy (DCM).

Rank	Organizations	Documents	Citations
1	Jilin University	44	2,019
2	Wenzhou Medical University	43	1,966
3	University of Louisville	40	2,021
4	Shandong University	23	968
5	Wuhan University	17	468
6	Harbin Medical University	17	796
7	Monash University	11	444
8	China Medical University	11	387
9	Charité	8	797
10	Huazhong University of Science and Technology	8	257

### Authors

**FIGURE 4:**
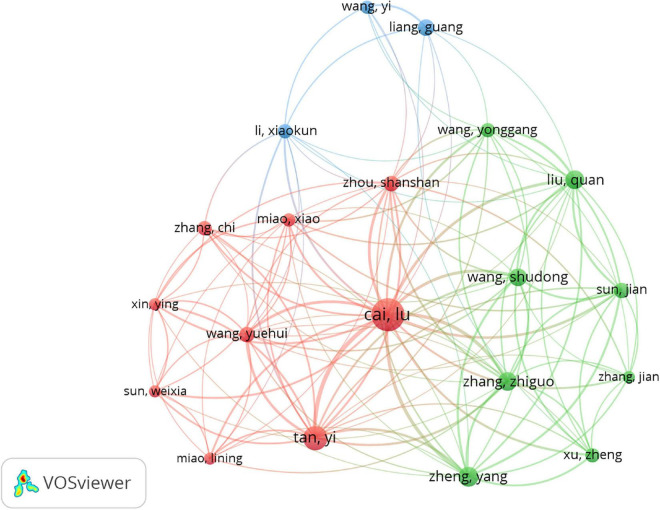
Network map of co-authorship analysis of authors in inflammation related to diabetic cardiomyopathy (DCM) research. The analysis method was Linlog/modularity in VOSviewer, the weight was an occurrence, and scores were the average published year.

The network map of co-authorship analysis of authors is constructed in Figure 4, and 42 satisfied the criterion (minimum number of documents of a keyword: 5). Table 4 lists the top 10 core authors in the field, with more than or equal to nine publications per author. Lu Cai was the first with 39 documents and had the highest citations (*n* = 1,988), followed by Yi Tan (21/1,322), Yang Zheng (14/765), Zhiguo Zhang (13/608), Quan Liu (12/507), Carsten Tschoepe (11/1,225), Shudong Wang (11/692), Liang, Guang (10/474), Heinz-peter Schultheiss (9/1,196), and Yuehui Wang (9/597). Though Carsten Tschoepe and Heinz-peter Schultheiss from Germany published fewer papers than other authors from China, they usually had higher citations. The results suggested that Lu Cai was the top scientist in this field.

**TABLE 4:** Core authors on inflammation in diabetic cardiomyopathy (DCM).

Rank	Authors	Country	Documents	Citations
1	Cai, Lu	United States	39	1,988
2	Tan, Yihui	United States	21	1,322
3	Zheng, Yang	China	14	765
4	Zhang, Zhiguo	China	13	608
5	Liu, Qian	China	12	507
6	Tschöpe, Carsten	Germany	11	1,225
7	Wang, Shudong	China	11	692
8	Liang, Guang	China	10	474
9	Schultheiss, Heinz-peter	Germany	9	1,196
10	Wang, Yuehui	China	9	597

### Co-occurrences of keywords

**FIGURE 5:**
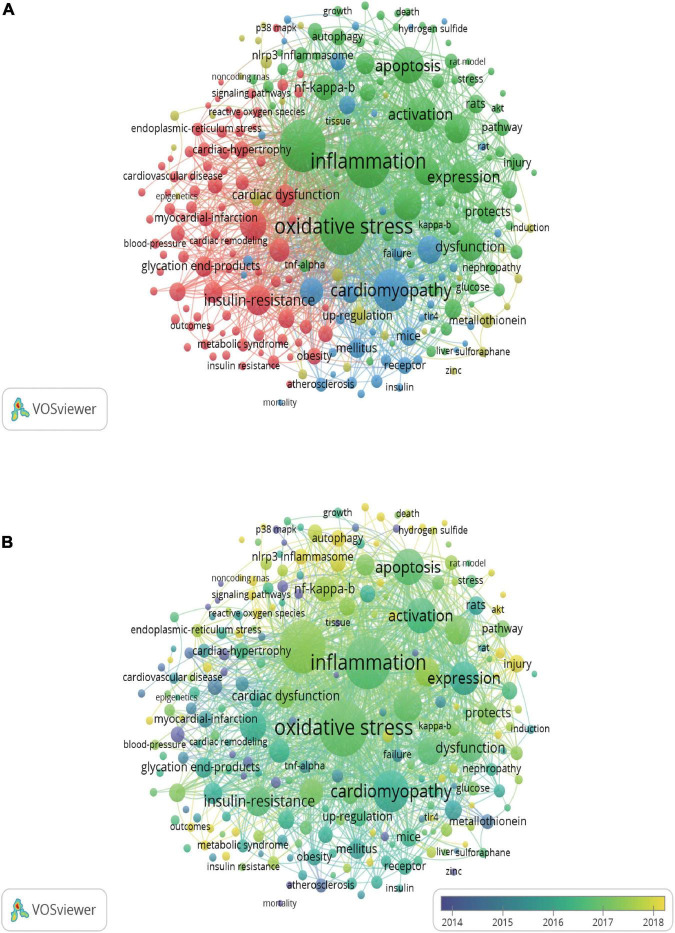
Network map of co-occurrence analysis of all keywords. **(A)** Mapping of keywords of publications. **(B)** Distribution of keywords according to average publication year. The analysis method was Linlog/modularity in VOSviewer, the weight was an occurrence, and scores were the average published year.

A total of 2,321 keywords were involved in 578 documents, and 234 satisfied the criterion (minimum number of documents of a keyword: 5). The network visualization map was constructed for the co-occurrence of all keywords (Figure 5A). The size of the circle indicates occurrences of keywords. Oxidative stress, inflammation, DCM, cardiomyopathy, apoptosis, activation, fibrosis, expression, and heart failure were high-frequency keywords. VOSviewer also colored the keywords according to the average appearing year (Figure 5B). Keywords that appeared relatively earlier were blue, and keywords with a more recent appearance were yellow. These keywords were published sequentially from 2014 to 2018 in this field. According to the statistical analysis of the author keywords, numerous molecules such as NF-κB, NLRP3 inflammasome, Nrf-2, TNF-α, protein kinase C (PKC), PPARα, TLR4, p38 mitogen-activated protein kinase (MAPK), TGF-β (transforming growth factor-β), Sirt1, and AKT were associated with inflammation in DCM. In addition, stem cells, fibroblasts, cardiomyocytes, other types of cells and apoptosis, oxidative stress, autophagy, endoplasmic reticulum (ER) stress, hypertrophy, mitochondrion dysfunction, and proliferation participated in the processes of inflammation in DCM. Some drugs such as sulforaphane, metformin, empagliflozin, and rosuvastatin were used to treat DCM based on targeting inflammation.

### Citation and co-citation

**FIGURE 6:**
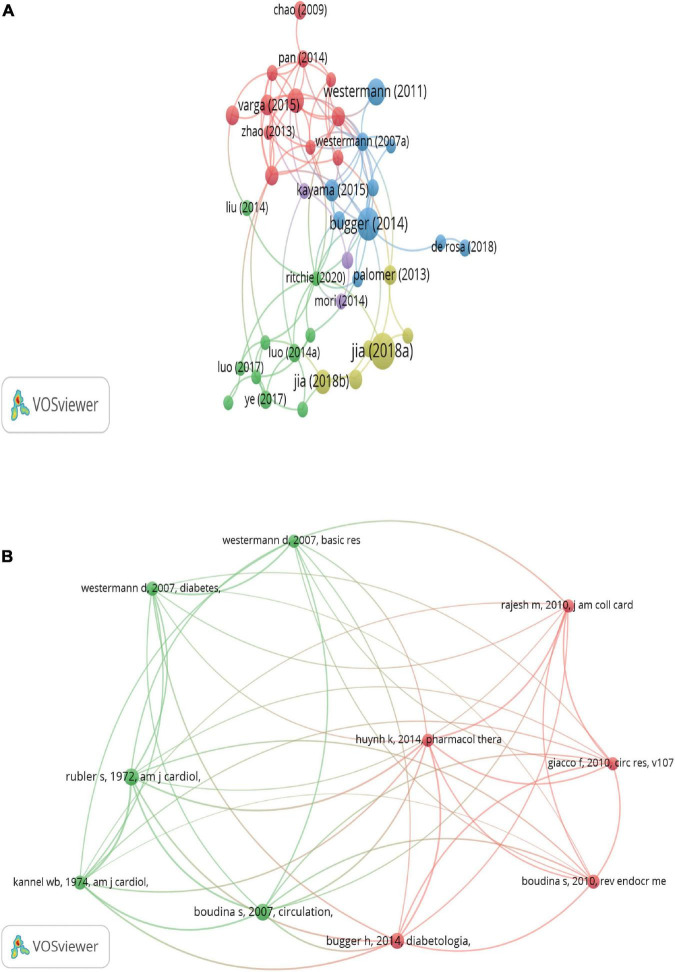
Network map of citation analysis of publications. **(A)** Mapping of citation analysis of documents. **(B)** Network map of co-citation analysis of references. The analysis method was Linlog/modularity in VOSviewer, the weight was an occurrence, and scores were the average published year.

Figure 6A shows the visualized network map of citations, and 50 satisfied the criterion (minimum number of documents of a keyword: 100). Table 5 lists the top night documents with the highest citations, each of which was more than 203, with a range of citations from 203 to 724. “Advanced Glycation End Products and Diabetic Complications,” published by Parkash Singh Varun, was cited 724 times and ranked the first, and “Interplay of oxidative, nitrosative/nitrative stress, inflammation, cell death and autophagy in diabetic cardiomyopathy” published by Zoltán V Varga was cited 203 times and ranked last among this ranking. Those highly cited documents focused on the role of inflammation in cardiovascular, particularly in DCM, and most were reviews.

**TABLE 5:** Top night citation analysis of publications on inflammation in diabetic cardiomyopathy (DCM) research.

Rank	Title	First author	Journals	Publication year	Citations
1	Advanced Glycation End Products and Diabetic Complications	Singh Varun, Parkash	The Korean Journal of Physiology & Pharmacology	2014	724
2	Diabetic Cardiomyopathy: An Update of Mechanisms Contributing to This Clinical Entity	Jia, Guanghong	Circulation Research	2018	659
3	Molecular mechanisms of diabetic cardiomyopathy	Bugger, Heiko	Diabetologia	2014	526
4	Cardiac Inflammation Contributes to Changes in the Extracellular Matrix in Patients With Heart Failure and Normal Ejection Fraction	Westermann, Dirk	Circulation: Heart Failure	2011	357
5	Diabetic cardiomyopathy: a hyperglycaemia- and insulin-resistance-induced heart disease	Jia, Guanghong	Diabetologia	2018	296
6	Cannabidiol Attenuates Cardiac Dysfunction, Oxidative Stress, Fibrosis, and Inflammatory and Cell Death Signaling Pathways in Diabetic Cardiomyopathy	Mohanraj Rajesh	Journal of the American College of Cardiology	2010	292
7	Diabetic Cardiovascular Disease Induced by Oxidative Stress	Kayama, Yosuke	International Journal of Molecular Sciences	2015	249
8	Role of Poly (ADP-ribose) polymerase 1 (PARP-1) in Cardiovascular Diseases: The Therapeutic Potential of PARP Inhibitors	Pacher Pál	Cardiovascular Drug Reviews	2007	233
9	Interplay of oxidative, nitrosative/nitrative stress, inflammation, cell death and autophagy in diabetic cardiomyopathy	Varga, Zoltán V	Biochimica et Biophysica Acta	2015	203

Figure 6B lists the top 10 references that were co-cited in more than 51 citations. The reference with the largest number of citations was published by Sihem Boudina (2007, circulation; 84 citations), followed by S Rubler (1972, American Journal of Cardiology, 83 citations), Heiko Bugger (2014, Diabetologia, 74 citations), Sihem Boudina (2010, Reviews in Endocrine and Metabolic Disorders; 60 citations), Dirk Westermann (2007, Diabetes, 56 citations), W B Kannel (1974, American Journal of Cardiology, 56 citations), Ferdinando Giacco (2010, Circulation Research, 54 citations), Mohanraj Rajesh (2010, Journal of the American College of Cardiology, 52 citations), Karina Huynh (2014, Pharmacology & therapeutics, 51 citations), and Dirk Westermann (2007, Basic Research In Cardiology, 51 citations).

## Discussion

### General trends in inflammation related to DCM

In the present study, we used bibliometric analyses and network visualizations to investigate the research trends of inflammation in DCM during the past two decades, which bring deep understanding to researchers in this field. The contributions of journals, authors, organizations, and countries to this emerging field were analyzed. Continued research interest during the two decades indicated that inflammation in DCM was a hot topic. With a steady increase in the number of annual publications and citations, more and more researchers are interested in inflammation related to DCM, which contributed to the growth of publications. China, the United States, and Germany were the top three countries, indicating their influences on the inflammation of DCM. Chinese scientists published the largest number of papers of all the identified articles. Eight of the top ten institutions in this field in terms of the number of publications were from China. However, two of the top ten core authors on inflammation in DCM were from Western countries. Hence, the analysis of research trends shows that authors from China and Western countries dominated the quality of publications on inflammation in DCM.

### Core journals and authors in inflammation related to DCM

As for the core journals in this field, the difference in papers was small, suggesting that all of them had considerable influence in this field. These journals regarding Diabetes and comprehensive journals preferred to publish these studies. Indeed, DCM was considered a common complication of diabetes (17). These journals were more likely to publish future developments in inflammation related to DCM. Journals concerning cardiovascular disease might also further contribute to this field in the future.

Based on the document analysis of core authors, Lu Cai and Carsten Tschöpe groups lead this field and contribute to the rapid progress. Lu Cai and his important collaborators (Yi Tan, Yang Zheng, and Zhiguo Zhang) were working on the role of metallothionein and Nrf2 signaling pathways in inflammation and oxidative stress associated with DCM (18–21). Furthermore, Lu Cai and Shudong Wang identified that metallothionein is downstream of Nrf2 (22). Lu Cai also identified some protective drugs for DCM treatment, such as sulforaphane, 4-O-methylhonokiol, and dimethyl fumarate, and revealed the mechanisms (23–25). Shudong Wang and Lu Cai Zinc prevented an inflammatory response in DCM (26, 27). Quan Liu and Guang Liang, cooperating with Lu Cai, mainly determined that JNK2 activation enhanced inflammation and DCM, while treatment with a novel curcumin derivative, C66, ameliorated DCM (28–30). Guang Liang further found that inhibition of the EGFR-STAT3 signaling pathway attenuated inflammation and DCM (31–33). He also elucidated some key molecules, such as MD2 and FGF1 (34, 35). In 2020, in collaboration with Yi Tan and Zhiguo Zhang, Lu Cai summarized the mechanisms of DCM and potential therapeutic strategies from preclinical and clinical evidence (36). Carsten Tschöpe cooperating with Dirk Westermann and Heinz-Peter Schultheiss verified the protective effects of the kallikrein-kinin system in the process of inflammation and DCM pathogenesis (37–39). They also reported that matrix metalloproteinases resulted in the crosstalk of inflammation and fibrosis involved in DCM (40–42). The literature analysis showed that the two groups lacked communication at present. In the future, more exchanges and cooperation between them might greatly promote developments in this field. The citation analysis of documents and co-cited analysis of references showed that these highly cited papers were reviews of DCM or inflammation in DCM, indicating little key progress was made in this field. Therefore, there is still great potential for development in this field.

### Keywords of inflammation in DCM

#### The pathophysiology and cell types in DCM

Currently, more and more scientists focus on the key role of inflammation in DCM. Inflammation was regarded as a trigger of DCM (43). Co-occurrences of keyword analysis suggested that the interaction between inflammation and oxidative stress causes cardiomyocyte death, cardiac hypertrophy, fibrosis, and heart failure in DCM (44). The proinflammatory cells, especially macrophages, play a key role in the development of DCM (45). Macrophage M1 phenotype triggers inflammation and DCM (46). Mesenchymal stem cells inducing type 2 macrophage polarization ameliorate DCM (47). Activation and expression of proinflammatory cytokines, such as TNF-α, interleukins (IL)-1β, -6, and -18, monocyte chemotactic protein 1 (MCP-1), and adhesion molecule intercellular adhesion molecule 1, contribute to cardiac oxidative stress, cardiac remodeling, and fibrosis, and diastolic dysfunction (48, 49). The activation and interaction of these types of cells govern the development of DCM.

#### The molecules regulating inflammation in DCM

Based on the co-occurrence of keywords analysis, many signaling pathways were associated with inflammation in DCM, and these interventions targeting them had been reported to reduce inflammation and attenuate DCM. NF-κB pathway activation follows a transphosphorylation chain from the specific activated receptors to the IκB proteins, which triggers NF-κB heterodimer to enter the nucleus and controls pro-inflammation gene expression, contributing to DCM (50). Hyperglycemia-induced p38 MAPK activation promotes inflammation and DCM (51). Fibroblast growth factor 21 (FGF 21)/SIRT1 pathway mediated autophagy inducing inflammation and DCM (52).

According to the core authors’ analysis and their contribution, some other important molecules in inflammation and DCM were also identified, such as the kallikrein-kinin system (38) and EGFR-STAT3 (33). PKC inhibitions have previously been linked to the decrease in the expression of inflammatory cytokines, facilitating cardiac damage and interstitial fibrosis *in vivo* and *in vitro* (53). In DCM, AGEs (advanced glycation end products) bind directly to MD2, leading to the formation of AGEs-MD2-TLR4 complexes and the initiation of pro-inflammatory pathways (34). DCM was associated with diminished Akt phosphorylation, which might contribute to myocardial dysfunction, cardiac fibrosis, inflammation, and interrelated signaling pathways (54). As was well-known, TGF-β served as a key mediator of fibrosis. The activation of inflammation enhances the synthesis of extracellular matrix (ECM) *via* upregulation of TGF-β resulting in diabetic cardiac dysfunction (55). Though numerous pathogenic targets and therapeutic targets of DCM have been found, effective therapeutic strategies for DCM remain elusive. Exploring the complex inflammatory response mechanism in the development and progression of diabetic cardiomyopathy is significant, which will inform more therapeutic strategies and clinical progress.

#### Therapeutic drugs against inflammation in DCM

The SGLT2 inhibitor empagliflozin is a novel drug against inflammation, fibrosis, and antioxidative stress in DCM (56). Rosuvastatin and other statins have been proven to attenuate inflammation and DCM *via* NLRP3 inflammasome and MAPK signaling pathways (57). Sulforaphane reduced cardiac oxidative damage, inflammation, fibrosis, and hypertrophy through the Nrf2 pathway (21). Metformin possessed cardioprotective and anti-inflammatory effects by activating AMPK/autophagy and inhibiting the NLRP3 inflammasome in DCM (58).

### Strengths and limitations

To the best of our best knowledge, the present study provided a deep insight into the global status and trends of research on inflammation in DCM using bibliometric analysis for the first time. Bibliometric analysis is objective, comprehensive, and widely used contributing to the reliability of the data. However, some limitations, which were commonly similar to other bibliometric analyses, should be addressed. First, the deadline for publications was 28 February 2021, but WOS Core Collection would also keep updating. Newly published documents have already been online on the websites of journals, and this part was lacking in this manuscript. Therefore, the data in this work could not fully reflect the reality of trends in this field. Second, this study was limited to WOS Core Collection indexed journal, and a few papers that were not in the scope of the inclusion were missed. Third, the terms “inflammation,” “diabetic cardiomyopathy,” “Article,” and “Reviews,” which appeared in the title, abstract, and keywords, were selected for this manuscript, while the related terms in the text could not be retrieved and analyzed. The results of keyword analysis might have been affected by incomplete keyword extraction. Despite these limitations, this study provides a solid global view of inflammation in DCM research over the last two decades.

## Conclusion

We used bibliometric analysis to provide a comprehensive overview of the research status of inflammation in DCM worldwide over the past two decades. This field is undergoing a period of rapid development and attracting the attention of an increasing number of researchers. China, the United States, and Germany made the most contributions. These journals regarding diabetes are productive in this field. Lu Cai and Yihui Tan were critical groups in the field of inflammation in DCM. Critical factors associated with inflammation in DCM, such as pathophysiology, various types of cells, inflammatory factors, key signaling pathways, and drugs, are hot topics in this research field. Our study provides a valuable reference for researchers, and the topics regarding inflammation in DCM deserve continued following up by researchers.

## Data availability statement

The original contributions presented in this study are included in the article/supplementary material, further inquiries can be directed to the corresponding author.

## Author contributions

NZ designed this study and revised the manuscript. BH and LZ performed the search, wrote the manuscript, and contributed to the data collection and verification. All authors read and approved the final manuscript.
